# Smartwatch User Authentication by Sensing Tapping Rhythms and Using One-Class DBSCAN

**DOI:** 10.3390/s21072456

**Published:** 2021-04-02

**Authors:** Hanqi Zhang, Xi Xiao, Shiguang Ni, Changsheng Dou, Wei Zhou, Shutao Xia

**Affiliations:** 1 Tsinghua Shenzhen International Graduate School, Shenzhen 518000, China; zhq19@mails.tsinghua.edu.cn (H.Z.); xiaox@sz.tsinghua.edu.cn (X.X.); ni.shiguang@sz.tsinghua.edu.cn (S.N.); xiast@sz.tsinghua.edu.cn (S.X.); 2 School of Statistics, Capital University of Economics and Business, Beijing 100000, China; 3 School of Software and Electrical Engineering, Swinburne University of Technology, Melbourne 3000, Australia; weizhou09@gmail.com

**Keywords:** one-class classification, DBSCAN, smartwatch, tapping rhythm, sensor, authentication

## Abstract

As important sensors in smart sensing systems, smartwatches are becoming more and more popular. Authentication can help protect the security and privacy of users. In addition to the classic authentication methods, behavioral factors can be used as robust measures for this purpose. This study proposes a lightweight authentication method for smartwatches based on edge computing, which identifies users by their tapping rhythms. Based on the DBSCAN clustering algorithm, a new classification method called One-Class DBSCAN is presented. It first seeks core objects and then leverages them to perform user authentication. We conducted extensive experiments on 6110 real data samples collected from more than 600 users. The results show that our method achieved the lowest Equal Error Rate (EER) of only 0.92%, which was lower than those of other state-of-the-art methods. In addition, a statistical method for detecting the security level of a tapping rhythm is proposed. It can prevent users from setting a simple tapping rhythm password, and thus improve the security of smartwatches.

## 1. Introduction

There are various types of smart sensing systems, including smart glasses, earphones, fitness wristbands, smartwatches, etc. [1]. With the development of technology, more and more smart systems are used in our lives. For example, since Motorola released the Moto 360 with Android Wear as its operating system in March 2014, fitness bands and smartwatches have become increasingly popular in recent years. Most of a user’s physiological data that are measured by the body sensors of a smart system are personal information, which is private and more sensitive than mobile phone numbers or email addresses [2]. Malicious people or organizations can infer high-value intelligence from the sensor data, such as the user’s identity, location, and health condition. Therefore, it is crucial to protect the security of these smart devices. Many efforts are being made towards lightweight security solutions tailored for smart sensing systems [3]. However, there is still a gap between the technology and the law, and we lack a standard approach to protecting equipment privacy [4,5,6]. It was reported by the U.S. Food and Drug Administration that more than 300 wearable devices from 40 manufacturers have privacy and security risks [7,8]. If cloud computing technology is applied to protect privacy, it cannot respond to urgent requests, and there are also communication security problems. In edge computing, the computing and storage nodes are placed at the internet’s edge, which can address concerns such as latency and bandwidth costs [9,10]. The edge nodes can perform calculations themselves, rather than transmitting the data to the server for the calculation, which can reduce the risk of privacy leaks from edge nodes. Since smartwatches can be the edge nodes of the internet of things, edge computing can protect the privacy of these devices.

A survey on Implantable Medical Devices (IMDs) and the security of wearable devices [11] classified attacks into three categories, namely communication channel attacks (e.g., Bluetooth sniffing attacks), hardware attacks (e.g., those caused by accessing data through hardware addresses), and software attacks (e.g., malicious programs). In addition, the data of a device without a lock screen or user authentication function are easy to steal if the device is lost or secretly used by others. Researchers from HP evaluated the ten most advanced smartwatches, but found that only 50% of them have a lock screen function. Thus, the long-neglected user authentication for smartwatches should be treated with greater importance [12].

As more attention has recently been paid to user authentication, researchers have proposed various edge computing authentication methods, which mainly include the following three types. The first type of authentication method is based on the user’s physiological biometrics, such as the relevant attributes of the eyes [13,14], Electrocardiogram (ECG), and Electroencephalogram (EEG) [15,16], as well as the body’s feedback of external stimuli [17,18]. Specific sensors should be included in smartwatches with these methods. However, commercial smartwatches are usually not equipped with these sensors [19]. The second authentication type is based on behaviors, such as walking postures [20,21]. Refs. [22,23,24,25,26] predicted when users performed specific actions according to the values of accelerometer sensors. These methods do not require user intervention; thus, the smartwatch automatically recognizes the user. The sampling frequency should be above 50 Hz [23] to achieve higher accuracy. However, authentication with a sampling frequency above 50 Hz at all times results in high energy consumption. In addition, in order to achieve higher accuracy, the duration of the gesture is preferably more than five seconds [23], which may embarrass users in public. The third type is comprised of knowledge-based authentication methods, such as passwords and patterns. However, it is very inconvenient to type passwords or draw patterns on a smartwatch due to its small screen and keypad. Moreover, even if a password is set, users tend to choose a simple and fast-unlocking one, since they usually unlock the screen, which makes it less secure [23]. In addition, passwords and patterns are vulnerable to thermal attacks or smudge attacks [23]. Thus, passwords are not suitable for smartwatches.

A new knowledge-based authentication method was proposed by Ben Hutchins et al. [27], which recognizes users by their tapping rhythms. The average verification time is only 1.7 s, which is lower than that of the pattern password (4.5 s) and that of the gesture password (16.5 s) [27]. The power consumption is only 181.4 mW, which is similar to that of screen opening (161.5 mW) [27]. In addition, the False Acceptance Rate (FAR) of the zero-effect attacks on the tapping rhythm is only 8.2% [27]. Thus, the tapping rhythm has the advantages of a short verification time, less power consumption, and strong robustness. Moreover, the tapping rhythm can be applied to user identification of small devices, since the tapping only occupies a small area. However, the average Equal Error Rate (EER) in Ben Hutchins et al. [27] is 7.2%. Even if we reproduce their method on our datasets, the lowest EER reaches 4.04%, which means that the important privacy of 4 of 100 people is at risk of leakage.

In this paper, we propose a new method for authentication of users based on edge computing by smartwatches via tapping rhythm analysis, which can decrease the EER from 4.04% to 0.92%. Further, a new classification algorithm called One-Class DBSCAN is presented to recognize users. Based on clustering, One-Class DBSCAN seeks core objects of the training data and leverages them to classify new samples. Our method outperforms another authentication approaches that use tapping rhythms [27]. In addition, there are mechanisms for detecting the security levels of text passwords, e.g., to see whether a password contains uppercase letters or not. However, to the best of our knowledge, there are no detection methods for tapping rhythms. Therefore, based on the standard deviation, we propose an approach to detecting the security of tapping rhythms. This can improve the security of tapping rhythm passwords by prompting users with the security level.

Two main contributions of this paper are as follows:

1. A new one-class classification algorithm called One-Class DBSCAN is proposed, which contributes a solution to the one-class classification.
2. We also propose a method that can detect the security level of a tapping rhythm and prompt users to set more complex passwords if the password is too simple.

In Section 2, related works on user authentication are presented. Section 3 describes a new methodology. The experimental datasets and some evaluation results are explained in Section 4. In Section 5, we introduce a method for detecting the security of a tapping rhythm. Section 6 relates to conclusions and future plans.

## 2. Related Work

User authentication is a basic function for preventing unauthorized users from turning on a device. The ways of recognizing users can be partitioned into three categories, which are authentication based on physiological biometrics, authentication based on behaviors, and authentication based on knowledge [28,29,30].

### 2.1. Authentication Based on Physiological Biometrics

Authentication based on physiological biometrics takes the user’s physiological characteristics as user-specific attributes, among which the most common ones are the fingerprint and iris. Iris authentication has been used to identify users of smart glasses [13]. Wang et al. [14] proposed a method for pupil detection under different illuminations. However, applying iris or fingerprint authentication to a smartwatch with a small size and low power consumption would inevitably increase its size or hardware cost.

There are also many medical ways to recognize users. Dustin van der Haar et al. [15] presented a biometric identification method based on a hybrid attribute. They built a system named CaNViS to classify people by their Electrocardiogram (ECG) and the Electroencephalogram (EEG), which are the most common bio-signals in the medical field. Since everyone has a different ECG and EEG, this method is universal. ECG was also employed by Chun et al. [16] for user authentication. However, commercial smartwatches do not necessarily have these two biosensors.

In addition, some researchers distinguished different people according to the responses of their bodies. Based on the fact that biological tissue responds accordingly when an electric current is applied, Cornelius et al. [17] designed bioimpedance models to recognize users. A typical biometric identification system called VibID, which is based on arm vibration information, was constructed in Yang et al. [18]. This system identifies users by their different body tissue responses to mechanical vibrations owing to their different physical characteristics. However, the wearing position of the wristband has a great influence on the accuracy of this system, so it is not able to record the long-term changes in a user’s body.

### 2.2. Authentication Based on Behavioral Biometrics

Authentication based on behaviors makes use of the sensor data of certain actions by users. Many researchers have proposed novel authentication methods applied to computers and mobile terminals. Mare et al. [31] constructed a wristband device that recognized users by comparing the movement track of the wrist—automatically detected by the device—with the data that users inputted into a terminal. Ren et al. [32] applied thane accelerometer to a phone according to users’ unique gait patterns. Draffin et al. [33] identified users by detecting the location and area of pressing points, as well as the force of touch. Non-stop authentication based on users’ behavior of touching the screen was proposed by Frank et al. [34] and Luca et al. [35]. However, these approaches are only applicable to devices with large screens, such as computers and mobile phones.

Some researchers have proposed implicit authentication methods without user intervention for wristband devices. Kwapisz et al. [20] employed a wristband-type accelerometer to recognize users based on their actions, such as sitting, walking, and running. Likewise, Yunze Zeng et al. [21] also took the sensor data of movements, such as walking, running, climbing, and jumping, as features for identifying users. The authors of [20,21] intended to directly and automatically recognize users by their sensor data without the user’s intervention at all times. However, it resulted in high battery consumption.

In addition, some explicit authentication approaches have also been put forward. Junshuang Yang et al. [22] classified users by the values that sensors collect when users draw circles, lift the device, and lay it down. Chao Shen et al. [23] constructed a classifier for unlocking a smartwatch, which was based on acceleration sensor data when users waved their hands, and the EER of the classifier was 4.27%. Similarly, Wu et al. [24], Akl et al. [25], and Liu et al. [26] also employed three-axis acceleration to identify people based on their gestures. The response to external audio was detected to recognize users in the work of Li et al. [36]. However, Chao Shen et al. [23] showed in their research that, in order to achieve better accuracy, the duration of the gesture is preferably more than five seconds. Moreover, they presented that the sampling frequency should be above 50 Hz. These all result in high energy consumption. Furthermore, users might be reluctant to make such unusual movements in public. Thus, these methods do not befit most people.

### 2.3. Authentication Based on Knowledge

These methods recognize users according to some knowledge, such as a text password. The most common password is a four-digit PIN code, but it is too simple and not safe enough. Even if attackers do not know the user, they can still guess the PIN code. Text passwords that include English letters have higher security than four-digit PIN codes and are widely used in smartphones and smart computers, but it is very difficult for users to input passwords into a smartwatch due to its small screen.

Some researchers have proposed authentication methods for mobile phones based on tapping rhythms of the users. Vasaki Ponnusamy et al. [37] utilized x-coordinate, y-coordinate, pressure, size, and tapping time as features, and built a classifier using machine learning algorithms to recognize users. Satvik Kulshreshtha et al. [38] proposed Woodpecker, which is an authentication method that enables users to tap secret rhythms on the backs of mobile devices. However, neither of them were about smartwatch user authentication.

Ben Hutchins et al. [27] presented a method of user authentication for smartwatches according the rhythm of tapping on the screen. The rhythm length was the number of times that the device was tapped. Their study suggested that a rhythm of the same length as a text password would be safer and more difficult to for attackers to crack. The tap time, release time, intervals between two successive taps, and relative intervals were used as features in their method. The length of a tapping rhythm data vector was *N* (*N* times of tapping) and the number of features was 4N−3. The average and the standard deviation of all training data were calculated. The category of the new sample was determined based on its distance from the average. The average EER of their approach was 7.2%.

## 3. Methodology

We introduce the proposed method in this section, which mainly includes the feature extraction, model training, and authentication parts.

### 3.1. Feature Extraction

**Figure 1:**
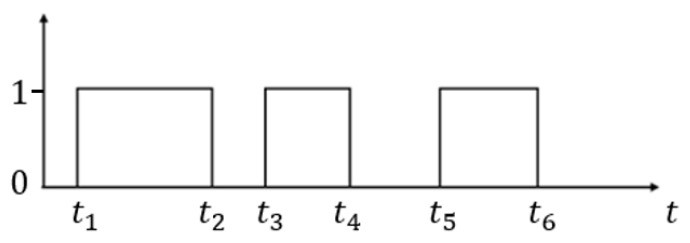
An example of one tapping rhythm data vector. The *x*-axis indicates the time and the *y*-axis indicates whether the screen is tapped (1 represents the screen being tapped, while 0 means that it is not).

One tapping rhythm datum is composed of many time instances of beats. For the convenience of description, the following tapping rhythm data vector is equivalent to a tapping rhythm datum. Figure 1 shows an example of one tapping rhythm data vector, in which the x-axis indicates the time and the y-axis indicates whether the screen is tapped. From the figure, we see that there are three durations, $t_{2}-t_{1}$, $t_{4}-t_{3}$, and $t_{6}-t_{5}$, and two intervals, $t_{3}-t_{2}$ and $t_{5}-t_{4}$. Thus, the tapping rhythm data vector can be described as a vector [$t_{2}-t_{1}$, $t_{3}-t_{2}$, $t_{4}-t_{3}$, $t_{5}-t_{4}$, $t_{6}-t_{5}$]. For a tapping rhythm data vector of *N* beats, the dimension of its feature vector is 2N−1.

Each element of the feature vector transformed from the tapping rhythm data represents one duration or interval. The overall duration of the rhythm is shorter if the user is in a state of urgency or excitement when tapping, and it may be longer if the user is in a leisurely mood. Therefore, there is a positive relationship between the total duration of the tapping rhythm and each duration or interval. Because of this, the feature vector can be transformed into the ratio of each duration or interval to the total duration of the tapping rhythm. As a result, the feature values of the vector remain almost unchanged regardless of if the user is in a state of leisure or urgency, which can increase the similarity of each input of the same tapping rhythm and thus improve the accuracy of the final judgment.

The function can be described by Equation (1), where $x=(x_{1},x_{2},\ldots ,x_{n})$ represents the feature vector, $x_{i}$ and $x_{j}$ are feature values, and $x_{i}^{\prime }$ is the transformed feature vector. *D* is the dimension of the vector, which is equal to 2N−1. The dimension of the transformed feature vector is the number of durations and intervals. The value of the vector can be described as the ratio of each duration or interval to the total duration of the tapping rhythm.

(1) $$x_{i}^{\prime }=\frac{x_{i}}{\sum_{j}^{D}x_{j}}$$

### 3.2. Model Training and Authentication

In traditional classification problems, the training datasets consist of a positive dataset and a negative dataset. After training, the model can predict whether a new sample is positive or negative. However, since there is only one class of the tapping rhythms inputted by the user, our work focuses on the one-classification issue. It is worth noting that, although the user has multiple favorite tapping rhythms, in our method, he/she can only set one as the authentication rhythm at each time (which is similar to how a user can only set one favorite password). One classification represents that the training dataset has only one class, and the classifier determines whether a new example belongs to this class. Since the training datasets for supervised learning usually have two classes, it is hard for us to utilize supervised learning algorithms to classify new samples in the one-classification issue. Since the clustering algorithm of unsupervised learning can cluster data without labels, it is adopted in our work. A new instance can be classified into one cluster. For the convenience of introduction, the formed cluster is equivalent to the class.

DBSCAN is a clustering algorithm proposed by Martin Ester et al. in 1996 [39]. The data vectors within the distance ϵ from the vector *u* constitute the neighbors of the vector *u*, which is denoted by $N_{\epsilon }\left(u\right)$. If $|N_{\epsilon }\left(u\right)|\geq MinPts$, the vector *u* is determined as a core object. Each core object stands for a cluster center. The vectors within the distance ϵ from the core object belong to the cluster of this core object. If the distance between core object *u* and core object *v* is less than ϵ, these two core objects belong to the same cluster. Thus, DBSCAN determines the core objects and the cluster by parameters MinPts and ϵ. Through calculation, the algorithm DBSCAN can find all core objects and all clusters. In addition, it is easier to satisfy the condition of the core object’s composition to determine more core objects if MinPts becomes smaller. Similarly, the number of vectors contained in one cluster increases as ϵ increases.

We propose a new classification algorithm called One-Class DBSCAN. One-Class DBSCAN generates only one cluster as the current class using all the training data. Algorithm 1 shows the algorithm of One-Class DBSCAN, whose main job is to calculate the core objects, and the cluster is defined based on the core objects. Feature extraction is illustrated in lines 1 to 5, and is described in Section 3.1. The Euclidean distance between each two data points is calculated to get the neighbors of each piece of training data, which are shown in lines 8 to 14. The training data whose neighbors are greater than MinPts, i.e., $|N_{\epsilon }\left(u\right)|\geq MinPts$, are regarded as the core object and are added to the set of core objects. Afterward, if there is no core object, the center of the training datasets is taken as the core object, as described in lines 19 to 25 of Algorithm 1. After that, One-Class DBSCAN obtains all core objects, which all belong to one class. The data vectors within the distance ϵ from the vector *u* constitute the neighbors of the vector *u*. If the number of neighbors is greater than MinPts, the vector *u* is determined as the core object. The core object is decided by the nearby data vectors. In general, since the outlier is far from the other normal data, the abnormal data are not judged as the core objects. Thus, the outlier cannot affect the performance of One-Class DBSCAN.

**Figure 2:**
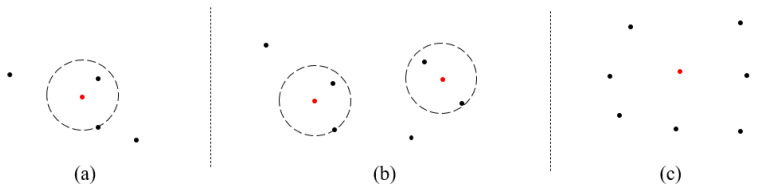
Examples of One-Class DBSCAN training.

Figure 2 illustrates several training examples. Red dots stand for the core objects, circles for the range where the distance from the core object is ϵ, and black dots for data. There is only one core object in Figure 2a; therefore, the two black dots in the circle belong to the class of the core object. In Figure 2b, the core objects and the black dots in their circles belong to the same class. There is no core object in Figure 2c, and thus, the average value of all data is employed as the core object.

After that, a new instance can be classified according to its distance ϵ from the core objects. Algorithm 2 demonstrates the authentication of One-Class DBSCAN. Similarly, it does feature extraction first in lines 1 to 3. Then, assuming there are *L* core objects, the algorithm traverses each core object and calculates the Euclidean distance from the new sample. If the Euclidean distance between the new sample and any core objects is less than ϵ, the sample is classified as a positive class, i.e., the class of training data, which means that the new sample passed the authentication.
**Algorithm 1** One-Class DBSCAN**Input:**  *D*: The dimension of the vector.  *m*: The size of the training data.  {$x^{\left(1\right)},x^{\left(2\right)},\ldots ,x^{\left(m\right)}$}: Training dataset  ϵ: The parameter of One-Class DBSCAN, which means that the distance is ϵ.  MinPts: The parameter of One-Class DBSCAN, which is the minimum number of data vectors within the distance ϵ required to form a core object.**Function:**1:**for**i=1,2,…,m**do**2: **for** j=1,2,…,D **do**
3:  $x_{j}^{\left(i\right)}=x_{j}^{\left(i\right)}/\sum_{k}^{D}x_{k}^{\left(i\right)}$
4: **end for**5:**end for**6:Initialize the set of core objects Ω=∅7:**for**i=1,2,…,m**do**8: $N_{\epsilon }\left(x^{\left(i\right)}\right)=\emptyset$
9: **for** j=1,2,…,m **do**
10:  $dis=\|x^{\left(i\right)}-x^{\left(j\right)}{\|}_{2}$
11:  **if** dis≤ϵ **then**
12:   $N_{\epsilon }\left(x^{\left(i\right)}\right)=N_{\epsilon }\left(x^{\left(i\right)}\right)\cup \left\{x^{\left(j\right)}\right\}$
13:  **end if**14: **end for**15: **if** $|N_{\epsilon }\left(x^{\left(i\right)}\right)|\geq MinPts$ **then**
16:  $\Omega =\Omega \cup \{x^{\left(i\right)}\}$
17: **end if**18:**end for**19:**if**Ω=∅**then**20: Initialize vector *v*21: **for** j=1,2,…,D **do**
22:  $v_{j}=\frac{1}{m}\sum_{k}^{m}x_{j}^{\left(k\right)}$
23: **end for**24: Ω = {*v*}25:**end if****Output:**  Ω: The set of core objects
**Algorithm 2** Authentication**Input:**  *D*: The dimension of the vector.  *L*: The number of core objects.  Ω: The set of core objects $\left\{\Omega^{\left(1\right)},\Omega^{\left(2\right)},\ldots ,\Omega^{\left(L\right)}\right\}$  ϵ: The parameter of One-Class DBSCAN, which means that the distance is ϵ.  *v*: The vector of the new sample.**Function:**1:**for**j=1,2,…,D**do**2: $v_{j}=v_{j}/\sum_{k}^{D}v_{k}$
3:**end for**4:**for**i=1,2,…,L**do**5: $dis=\|\Omega^{\left(i\right)}-v{\|}_{2}$
6: **if** dis≤ϵ **then**
7:  return True8: **end if**9:**end for**10:return False**Output:**  True or False: Whether the new sample belongs to this class

## 4. Experiment

In this section, experiments are conducted with the methods described in Section 3, and some evaluation results are presented. We evaluated the tapping rhythm program with an Android Virtual Device (AVD). The CPU of this AVD was the Wear OS Intel Atom (x86) with four cores, the Random Access Memory (RAM) size was 512 MB, the SD card size was 512 MB, and the Android API version was 28. We briefly introduce our datasets and evaluation indicators in Section 4.1, and the experimental process is presented in Section 4.2. After that, experimental results are given in Section 4.3 and Section 4.4. Finally, in Section 4.5, we measure the running time.

### 4.1. Datasets and Evaluation Indicators

To the best of our knowledge, there were no public datasets for tapping rhythms on smartwatches when we performed our evaluations. Thus, we collected data on a Moto 360. In order to record data for our method, we developed a program on an Android smartwatch. Each user inputs one favorite tapping rhythm 10 times (these 10 tapping rhythm data belong to the same class). When the user inputs the tapping rhythm, the program records the tapping rhythm data automatically in the smartwatch. After that, the collected datasets are transmitted to a PC. A total of 6110 pieces of data were collected from more than 600 people. The data lengths and the sizes of the datasets are shown in Table 1. The limited sizes of datasets with the length of N=9 and N=10 may lead to inaccurate experimental results; thus, we only utilized datasets with lengths of 5 to 8.

Three indicators, i.e., the False Acceptance Rate (FAR), False Rejection Rate (FRR), and Equal Error Rate (EER), were applied to evaluate the model. The FAR is the ratio of the number of supposititious data that are considered legitimate to the total number of supposititious essays [27], representing the ratio of impostors acknowledged by our proposed approach [23]. The FRR is the ratio of the count of incorrect authentications in legitimate data to the total count of legitimate attempts [27], indicating the ratio of legitimate users rejected by our proposed approach [23]. The effect of the classifier becomes better as the FAR and FRR decrease. However, with the adjustment of parameters, the FAR and FRR are generally inversely proportional. Thus, to balance it, we also take account of EER, which is the value where FRR = FAR [27]. Similarly, the smaller the EER, the better the classifier.

### 4.2. Implementation

Firstly, we carried out experiments with the proposed method described in Section 3 to evaluate the model. Further, the approach from Ben Hutchins et al. [27] was reproduced to compare with our method. Since the two methods to be compared have different features and classification algorithms, these two aspects were compared separately with two experiments for each. There were four experiments in total.

The evaluation process is shown in Algorithm 3. Each class is treated as a legitimate class, and the remaining classes of the same length are regarded as supposititious classes for cross-validation. Five random datasets in the legitimate class were chosen as the training data, while the remaining five datasets in the legitimate class and another five datasets selected randomly from the remaining classes were chosen as the testing data in every training and testing. This process was repeated 10 times to calculate the average mean. For various algorithm parameters, the FAR and FRR were different, but always inversely proportional. Thus, in order to balance the FRR and FAR, the model was evaluated with the equivalent value of the FAR and FRR, i.e., EER. Thus, the parameters at the EER are the optimal parameters. We took datasets of the same length together for cross-validation to obtain the EER and the optimal parameters. After that, we calculated the average EER from each length of data. Similarly, we calculated the average parameters from these optimal parameters to build a model with fixed parameters that can make predictions for datasets of all lengths.
**Algorithm 3** Evaluation process1:**for** 10 times **do**2: **for***c* in classes **do**3:  $c^{\prime }$ = classes except *c*4:  trainDataset = choose 5 data in *c* randomly5:  testDataset = remaining 5 data in *c*6:  testDataset += choose 5 data in $c^{\prime }$ randomly7:  Train model and calculate FAR,FRR8: **end for**9:**end for**10:Calculate the average mean of FAR, FRR11:Calculate EER

### 4.3. Ablation Study

**Figure 3:**
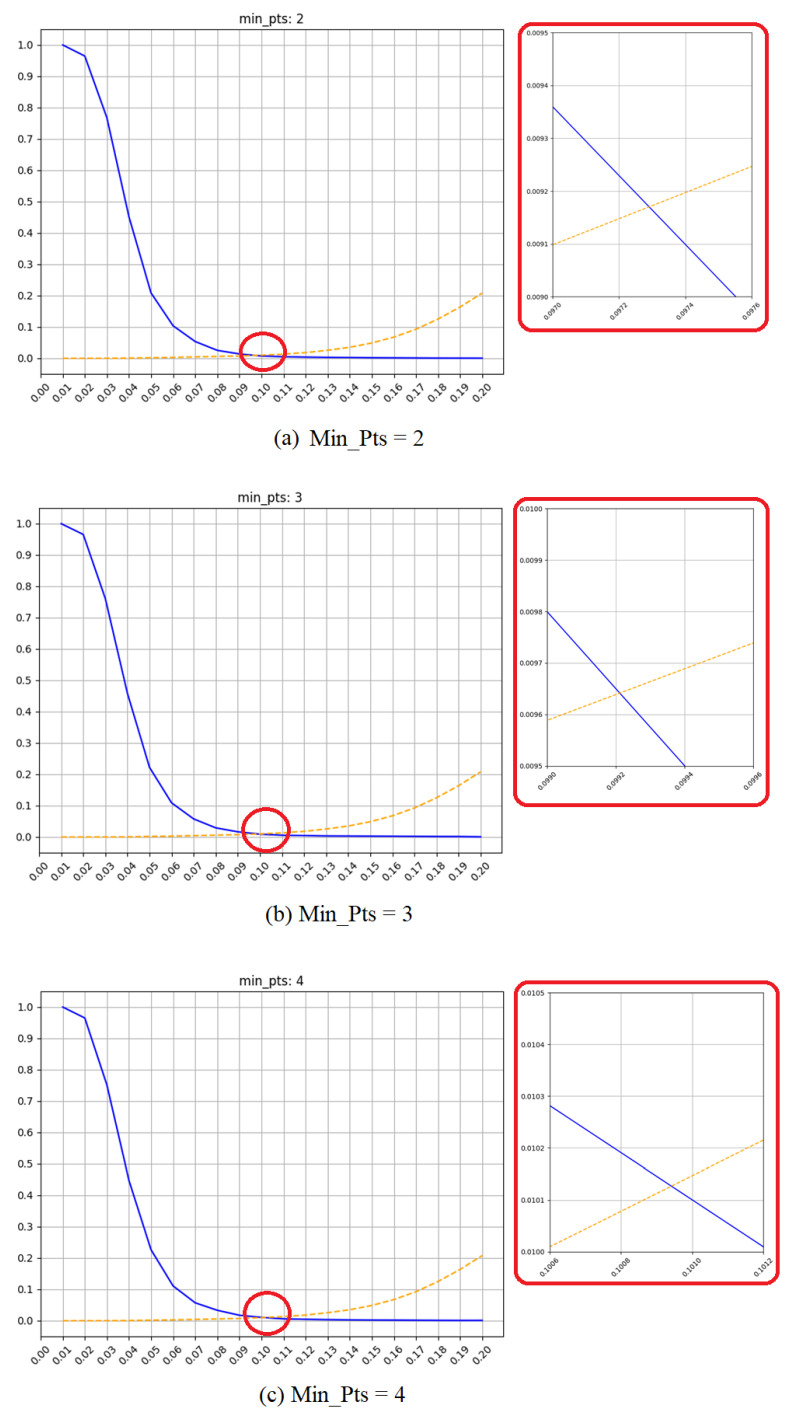
Results of One-Class DBSCAN. The blue is False Rejection Rate (FRR), and the orange dash is False Acceptance Rate (FAR).

As described in Section 3.2, two parameters were used to adjust One-Class DBSCAN. Thus, MinPts was limited to 2, 3, and 4, and ϵ was set from 0.01 to 0.20 to find the best combination of parameters. Figure 3 shows the results of the evaluation of One-Class DBSCAN. The blue and orange lines represent the FRR and FAR, respectively, which are inversely proportional. Intuitively, the parameters with low EER could always be found. The FAR became larger if ϵ was greater than 0.2, and the FRR approached 1 when ϵ was less than 0.01. Therefore, considering both the FAR and FRR, the optimal ϵ was between 0.01 and 0.2. The figure in the red box is an enlarged view at the lowest point of the original figure. Through the enlarged figure, we can find that the EER and optimal ϵ are slightly different when MinPts changes. The optimal ϵ and EER at different MinPts are illustrated in Table 2. It is clearly seen that MinPts has little effect on the experimental results, but the results of MinPts=2 are still slightly better than those of 3 and 4. Therefore, combining Figure 3 with Table 2, the optimal parameters are MinPts=2 and ϵ=0.0973.

Table 3 illustrates the experimental results for data of different lengths under the condition of MinPts=2 and ϵ=0.0973. Clearly, as the data length increases, the FRR and FAR become smaller. This means that the security of the password increases as the password length increases.

### 4.4. Comparison

In this section, we compare One-Class DBSCAN with the method of Ben Hutchins et al. [27], the clustering algorithm Mean Shift, the anomaly detection algorithm Isolation Forest, and two supervised learning algorithms. The method of Ben Hutchins et al. [27] was reproduced on our datasets as a base line. It employed the tap time, release time, intervals between two successive taps, and relative intervals as features to identify one piece of tapping rhythm data. Their classification algorithm was called Vector Comparison. The mean vector $\bar{f}$ and the standard deviation vector σ of the training datasets were utilized to build the model. If $\|f-\bar{f}{\|}^{2}\leq \alpha \|\sigma {\|}^{2}$, the new sample vector *f* was classified into the positive class. The optimal parameter (α=2.82) after adjustment and the corresponding EER (4.04%) of the method of Ben Hutchins et al. [27] are shown in Table 4. Obviously, our method (EER = 0.92%) outperformed that of Ben Hutchins et al. [27] (EER = 4.04%) on the real-world datasets. In order to better explain why our method is better than theirs, we performed further experiments, i.e., the experiments of comparing the different features and classification algorithms separately.

The different features and classification algorithm of our approach are innovative. To validate the effectiveness of our features, we replaced features of our method with those of Ben Hutchins et al. [27], and the results are shown in the row “Features from Ben Hutchins et al. [27] and One-Class DBSCAN” in Table 4. From the table, we see that after the parameter adjustment, the EER was 2.51%, which is higher than that of our method (EER = 0.92%), indicating that our features performed better than theirs. We believe that the features have a significant impact on the accuracy of the classification. If their features (“Features in Ben Hutchins et al. [27] and One-Class DBSCAN” in Table 4) are replaced by our features (“Our method” in Table 4), the EER can be reduced from 2.51% to 0.92%.

Afterward, we substituted the Vector Comparison algorithm from Ben Hutchins et al. [27] with One-Class DBSCAN to determine whether our algorithm is better. The row “Our features and Vector Comparison” in Table 4 shows that the Vector Comparison algorithm had an optimal parameter of α=2.31 and EER of 1.06%, which was slightly higher than that of our algorithm (EER = 0.92%), suggesting that our algorithm performs slightly better than theirs as well.

In addition, we compared One-Class DBSCAN with the Mean Shift clustering algorithm [40] and the Isolation Forest anomaly detection algorithm [41]. Mean Shift is a hill-climbing algorithm that involves shifting a kernel iteratively to a higher-density region until convergence. It leverages a Gaussian kernel $k\left(x\right)=\exp\left(-\frac{x^{2}}{2\sigma^{2}}\right)$; σ is one parameter of this algorithm. Every data vector is first assigned a weight according to the distance from the kernel center. At every iteration, the kernel is shifted to the weighted mean of all data. Mean Shift judges whether the new sample belongs to the positive class according to the distance between the new sample and the kernel center. The result is shown in the row “Mean Shift” in Table 4, where EER = 53.4%. The reason for the poor accuracy of Mean Shift may be that the kernel function is not suitable for low-dimensional data, such as a tapping rhythm. Isolation Forest is an unsupervised learning algorithm for anomaly detection. The algorithm builds subtrees by randomly selecting a feature and then randomly choosing a split value between the maximum and minimum values of this feature. The new sample is classified from the root node of the tree to the leaf node. If the path from the root node to the leaf node is short, the new sample may be abnormal. The parameter n_estimators denotes the number of subtrees, and contamination represents the proportion of outliers in the datasets. The result is shown in the last row of Table 4, where the EER is 30.6%. Therefore, tree-based classification algorithms are not suitable for tapping rhythm data.

Further, we made more comparisons with two supervised learning algorithms, i.e., Decision Tree [42] and Logistic Regression [43]. In Decision Tree, criterion represents the function for measuring the quality of a split (“gini” is Gini impurity), splitter is the strategy used to choose the split at each node (“best” is to choose the best split), max_depth is the maximum depth of the tree, and min_samples_split is the minimum number of samples required to split an internal node. We tried many parameters, but we could not decrease the FAR. The FAR was always greater than the FRR, so we could not get EER. Therefore, in Table 5, we just give the results of the FRR and FAR. After adjusting the parameters, the best result was FRR = 7.0% and FAR = 24.9%, which indicates that the model usually treated the negative samples as positive samples. In Logistic Regression, *C* represents the inverse of the regularization strength. The best results were FRR = 0.09% and FAR = 16.8%, which was better than those of Decision Tree. In our method, EER = 0.92% means that FRR = FAR = 0.92%. Thus, our method outperformed the Decision Tree in FRR and FAR, and was better than Logistic Regression in FAR. In supervised binary classification problems, the training datasets consist of a positive dataset and a negative dataset. However, in the real environment, we can only obtain the input data of the target user, i.e., only the positive data, without the negative data. Since there is only one class of tapping rhythms inputted by users, supervised models cannot be trained.

In summary, our approach was better than that of Ben Hutchins et al. [27] in both the features and the classification algorithm. The EER values obtained through experiments suggested that our features can distinguish the datasets more easily, and One-Class DBSCAN can cluster the datasets more clearly. Our method also outperformed the Mean Shift clustering algorithm and the Isolation Forest anomaly detection algorithm. The experimental results also show that MinPts=2 is better than 3 and 4, and the lowest EER of 0.92% can be obtained when MinPts=2 and ϵ=0.0973. However, even if the length of the data is only 5, our method can still achieve an FRR of 1.11% and FAR of 1.16%, as shown in Table 3, indicating that our approach can achieve precise results, even with a small amount of training data.

### 4.5. Running Time

Since the time that it takes to input the tapping rhythm is dependent on the users, it could not be evaluated. Therefore, we measured the running time of the model training after the user entered the tapping rhythm and the running time of authentication after the user inputted a new sample. In order to ensure the accuracy of experiments, we measured the running times of the model training with different lengths of tapping rhythms separately. There were five training samples for each training, and the experiments are executed 10 times to calculate the average running time.

Table 6 shows the running times of training and authentication. It is clear that both the running time of training and the running time of authentication increased with the lengths of the tapping rhythms, i.e., *N*. Thus, we conclude that tapping rhythms with a long length require more running time. From Table 6, the average running time of training was 49.1 ms, which is negligible for users. Even when the lengths of the tapping rhythms reached 8, the average running time was only 55.6 ms. The average running time of authentication was much shorter than that of training, which was 8.9 ms when N=5 and 12.6 ms when N=8. Then, the average training time on the entire dataset was 52.45 ms, and the average authentication time was only 10.58 ms. Therefore, clearly, our method runs fast, so the running time is negligible for users.

## 5. Tapping Rhythm Security Improvement

For text passwords, there is a system that can detect password security, and if the password we set is too simple, it prompts us with a message like “contains any two of numbers and uppercase or lowercase letters” so that we can change the password to increase the security. However, there are no uppercase or lowercase letters or numbers that can be quantified in a tapping rhythm. Moreover, there is currently little research in this field, and as far as we know, no one has proposed a method for detecting the security of tapping rhythms. If there is a system that can detect the security of a tapping rhythm inputted by users and prompt them to set a more complex one when the password is simple, the risk of being cracked will be decreased. Therefore, we propose an approach based on the standard deviation for detecting the security of the tapping rhythm to fill the gap in the field, which can improve the tapping rhythm’s security.

**Figure 4:**
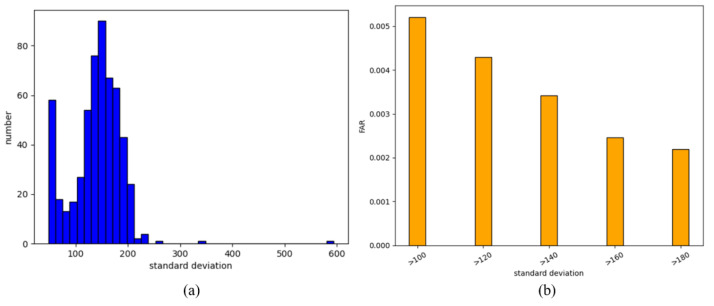
(**a**) Histogram of the standard deviation of the data. (**b**) FAR of different data with a standard deviation greater than $\sigma^{\prime }$.

We took all the data of one class as the training data and all the data of the remaining classes as the test data. The average standard deviation values of ten vectors in each class were obtained. Figure 4a illustrates the histogram of the standard deviation of the data, where the *x*-axis represents the standard deviation and the *y*-axis stands for the size of the data with the standard deviation within this interval. From the figure, we observe that the standard deviation of most data is concentrated in the interval of 100 to 200. To ensure the accuracy of the experimental results, we only compared the FAR and the standard deviation of the data with the standard deviation within 100 to 200.

We set a threshold for the standard deviation $\sigma^{\prime }$. In order to simulate the real environment, the data with a standard deviation below the threshold were not accepted. Then, we got the average FAR, which is shown in Figure 4b. The *x*-axis represents the threshold $\sigma^{\prime }$, and the *y*-axis stands for the average FAR. Clearly, it can be seen from the figure that the FAR decreases as the threshold $\sigma^{\prime }$ increases, demonstrating that the security of the password improves as the threshold increases. Thus, the standard deviation can be applied as a threshold to determine the FAR, which can be regarded as a criterion for detecting the security of the tapping rhythm password. From Figure 4, the threshold of the tapping rhythm can be set between 100 to 200.

## 6. Conclusions and Future Work

With the growing popularity of smart sensing systems, data security needs to be seriously taken into consideration. Existing user authentication methods for wearable devices have high power consumption, requirements of specific actions, and other disadvantages. In order to solve these problems, tapping rhythms were introduced to recognize individuals in this paper, and a new algorithm called One-Class DBSCAN was presented. The experimental results using real-world datasets showed that the lowest EER of our approach was only 0.92%, which indicates that the proposed method can effectively improve the security of smartwatches. The average training time of our method was 52.45 ms, and the average authentication time was only 10.58 ms. In addition, we proposed a method for improving the security of the tapping rhythm password by prompting users with the security level. The habit of tapping on a mobile device is unstable and changes as time goes on. We can periodically remind users to reset their tapping rhythm passwords to adapt to new habits. Further, incremental learning can be applied to update the model with the current tapping rhythms.

In the future, we will improve our method so that it can recognize multiple different tapping rhythms after one training (the user can set multiple favorite tapping rhythms at once). In addition, further investigation of user authentication for smart sensing systems will be conducted. Since our approach is flexible and feasible, it can be applied in many places with less data, and we will try to make breakthroughs in other application scenarios.

## Figures and Tables

**Table 1:** The sizes of tapping rhythm datasets.

*N*	5	6	7	8	9	10	Total
Num	1700	1770	1330	790	260	260	6110

**Table 2:** Equal Error Rate (EER) and optimal parameters of One-Class DBSCAN.

MinPts	2	3	4
ϵ	0.0973	0.0992	0.1009
EER	0.92%	0.96%	1.01%

**Table 3:** Experimental results when MinPts=2 and ϵ=0.0973.

*N*	5	6	7	8
FRR	1.11%	0.89%	0.85%	0.63%
FAR	1.16%	0.84%	0.83%	0.69%

**Table 4:** Comparison results.

Experiment	Optimal Parameter(s)	EER
Our method (Our features and One-Class DBSCAN)	MinPts=2, ϵ=0.0973	0.92%
Ben Hutchins et al. [27] method (Features in Ben Hutchins et al. [27] and Vector Comparison)	α=2.82	4.04%
Features in Ben Hutchins et al. [27] and One-Class DBSCAN	MinPts=2, ϵ=461.9	2.51%
Our features and Vector Comparison	α=2.31	1.06%
Mean Shift	σ=0.0418	53.4%
Isolation Forest	n_estimators=10, contamination=0.14	30.6%

**Table 5:** Experimental results using supervised learning algorithms.

Experiment	Optimal Parameter(s)	FRR	FAR
Decision Tree	criterion = gini, splitter = best, max_depth = 3, min_samples_split = 0.8	7.0%	24.9%
Logistic Regression	C=0.1	0.09%	16.8%

**Table 6:** The running times of training and authentication.

*N*	Training		Authentication	
	avg (ms)	std (ms)	avg (ms)	std (ms)
5	49.1	9.58	8.9	4.12
6	51.3	8.12	9.6	3.66
7	53.8	6.01	11.2	3.73
8	55.6	8.24	12.6	2.53
avg(ms)	52.45		10.58	

## Data Availability

Not applicable.

## References

[1] Seneviratne S., Hu Y., Nguyen T., Lan G., Khalifa S., Thilakarathna K. (2017). A Survey of Wearable Devices and Challenges. IEEE Commun. Surv. Tutor..

[2] Privacy Act 1988. Federal Register of Legislation. https://www.legislation.gov.au/Details/C2015C00279.

[3] Haghighi M.S., Nader O., Jolfaei A. (2020). A Computationally Intelligent Hierarchical Authentication and Key Establishment Framework for the Internet of Things. IEEE Internet Things Mag..

[4] Hoyle R., Templeman R., Armes S., Anthony D., Crandall D., Kapadia A. Privacy behaviors of lifeloggers using wearable cameras. Proceedings of the 2014 ACM International Joint Conference on Pervasive and Ubiquitous Computing.

[5] Saa P., Moscoso-Zea O., Lujan-Mora S. Wearable Technology, Privacy Issues. Proceedings of the International Conference on Information Technology & Systems.

[6] Stojmenovic I., Wen S., Huang X., Luan H. (2016). An Overview of Fog Computing and its Security Issues. Concurr. Comput. Pract. Exp..

[7] Sametinger J., Rozenblit J., Lysecky R., Ott P. (2015). Security Challenges for Medical Devices. Commun. ACM.

[8] Chen X., Li C., Wang D., Wen S., Zhang J., Nepal S., Ren K. (2020). Android HIV: A Study of Repackaging Malware for Evading Machine-Learning Detection. IEEE Trans. Inf. Forensics Secur..

[9] Satyanarayanan M. (2016). The emergence of edge computing. Computer.

[10] Weisong S., Schahram D. (2016). The Promise of Edge Computing. Computer.

[11] Yu J., Hou B. Survey on IMD and Wearable Devices Security Threats and Protection Methods. Proceedings of the International Conference on Cloud Computing and Security.

[12] Internet of Things Security Study: Smartwatches. https://www.ftc.gov/system/files/documents/public_comments/2015/10/00050-98093.pdf.

[13] Lee J.J., Noh S., Park K.R., Kim J. Iris Recognition in Wearable Computer. Proceedings of the International Conference on Biometric Authentication.

[14] Wang T., Song Z., Ma J., Xiong Y., Jie Y. An anti-fake iris authentication mechanism for smart glasses. Proceedings of the 2013 3rd International Conference on Consumer Electronics, Communications and Networks.

[15] van der Haar D. CaNViS: A cardiac and neurological-based verification system that uses wearable sensors. Proceedings of the Third International Conference on Digital Information, Networking, and Wireless Communications.

[16] Chun S.Y., Kang J.H., Kim H., Lee C., Oakley I., Kim S.P. ECG based user authentication for wearable devices using short time Fourier transform. Proceedings of the 39th International Conference on Telecommunications and Signal.

[17] Cornelius C., Peterson R., Skinner J., Halter R., Kotz D. A wearable system that knows who wears it. Proceedings of the 12th annual International Conference on Mobile Systems, Applications, and Services.

[18] Yang L., Wang W., Zhang Q. VibID: User identification through bio-vibrometry. Proceedings of the 15th International Conference on Information Processing in Sensor Networks.

[19] Wang Z., Shen C., Chen Y. Handwaving authentication: Unlocking your smartwatch through handwaving biometrics. Proceedings of the Chinese Conference on Biometric Recognition.

[20] Kwapisz J.R., Weiss G.M., Moore S.A. (2010). Activity Recognition using Cell Phone Accelerometers. ACM SIGKDD Explor. Newsl..

[21] Zeng Y., Pande A., Zhu J., Mohapatra P. WearIA: Wearable device implicit authentication based on activity information. Proceedings of the IEEE International Symposium on A World of Wireless, Mobile and Multimedia Networks.

[22] Yang J., Li Y., Xie M. MotionAuth: Motion-based authentication for wrist worn smart devices. Proceedings of the IEEE International Conference on Pervasive Computing and Communication Workshops.

[23] Shen C., Wang Z., Si C., Chen Y., Su X. (2020). Waving Gesture Analysis for User Authentication in Mobile Environment. IEEE Netw. Mag..

[24] Wu J., Pan G., Zhang D., Qi G., Li S. Gesture Recognition with a 3-D Accelerometer. Proceedings of the International Conference on Ubiquitous Intelligence and Computing.

[25] Akl A., Feng C., Valaee S. (2011). A Novel Accelerometer-Based Gesture Recognition System. IEEE Trans. Signal Process..

[26] Liu J., Zhong L., Wickramasuriya J., Vasudevan V. (2009). uWave: Accelerometer-based personalized gesture recognition and its applications. Pervasive Mob. Comput..

[27] Hutchins B., Reddy A., Jin W., Zhou M., Li M., Yang L. Beat-PIN: A User Authentication Mechanism for Wearable Devices Through Secret Beats. Proceedings of the Asia Conference on Computer and Communications Security.

[28] Shrestha P., Saxena N. (2018). An Offensive and Defensive Exposition of Wearable Computing. ACM Comput. Surv..

[29] Alzubaidi A., Kalita J. (2016). Authentication of smartphone users using behavioral biometrics. IEEE Commun. Surv. Tutor..

[30] Lin G., Wen S., Han Q.L., Zhang J., Xiang Y. (2020). Software Vulnerability Detection Using Deep Neural Networks: A Survey. Proc. IEEE.

[31] Mare S., Markham A.M., Cornelius C., Peterson R., Kotz D. ZEBRA: Zero-Effort Bilateral Recurring Authentication. Proceedings of the 2014 IEEE Symposium on Security and Privacy.

[32] Ren Y., Chen Y., Chuah M.C., Yang J. Smartphone based user verification leveraging gait recognition for mobile healthcare systems. Proceedings of the 2013 IEEE International Conference on Sensing, Communications and Networking.

[33] Draffin B., Zhu J., Zhang J. KeySens: Passive User Authentication through Micro-behavior Modeling of Soft Keyboard Interaction. Proceedings of the International Conference on Mobile Computing, Applications, and Services.

[34] Frank M., Biedert R., Ma E., Martinovic I., Song D. (2013). Touchalytics: On the Applicability of Touchscreen Input as a Behavioral Biometric for Continuous Authentication. IEEE Trans. Inf. Forensics Secur..

[35] De Luca A., Hang A., Brudy F., Lindner C., Hussmann H. Touch me once and i know it’s you! implicit authentication based on touch screen patterns. Proceedings of the SIGCHI Conference on Human Factors in Computing Systems.

[36] Li S., Ashok A., Zhang Y., Xu C., Lindqvist J., Gruteser M. Whose move is it anyway? Authenticating smart wearable devices using unique head movement patterns. Proceedings of the IEEE International Conference on Pervasive Computing and Communications.

[37] Vasaki P., Chan M.Y., Adnan B.A.A. Mobile Authentication Using Tapping Behavior. Proceedings of the International Conference on Advances in Cyber Security.

[38] Satvik K., Ahmed S.A. Woodpecker: Secret Back-of-Device Tap Rhythms to Authenticate Mobile Users. Proceedings of the 2020 IEEE International Conference on Systems, Man, and Cybernetics.

[39] Ester M., Kriegel H.P., Sander J., Xu X. A Density-Based Algorithm for Discovering Clusters in Large Spatial Databases with Noise. Proceedings of the Second International Conference on Knowledge Discovery and Data Mining.

[40] Comaniciu D., Meer P. (2002). Mean shift: A robust approach toward feature space analysis. IEEE Trans. Pattern Anal. Mach. Intell..

[41] Liu F.T., Ting K.M., Zhou Z.H. Isolation forest. Proceedings of the 2008 Eighth IEEE International Conference on Data Mining.

[42] Safavian S.R., Landgrebe D. (1991). A survey of decision tree classifier methodology. IEEE Trans. Syst. Man, Cybern..

[43] Pregibon D. (1981). Logistic regression diagnostics. Ann. Stat..

